# Supplementary material for: Unravelling the age of fine roots of temperate and boreal forests
Source: Nat Commun. 2018 Aug 1;9:3006. doi: 10.1038/s41467-018-05460-6 (PMC6070616; doi:10.1038/s41467-018-05460-6)
Supplement: Supplementary file 1 — Supplementary Information [file 41467_2018_5460_MOESM1_ESM.pdf]

Supplementary Information for

Unravelling the age of fine roots of temperate and boreal forests

Solly *et al*.

This file includes:

Supplementary Figures 1 to 2
Supplementary Table 1

Other Supplementary information for this manuscript includes the following:

Supplementary Data 1: Radiocarbon and ^{14}C age, Root biomass, In-growth cores, Tree seedling, Comparison of single roots (.xls).
Supplementary Software 1: R 3.2.3 code used for the statistical analysis.

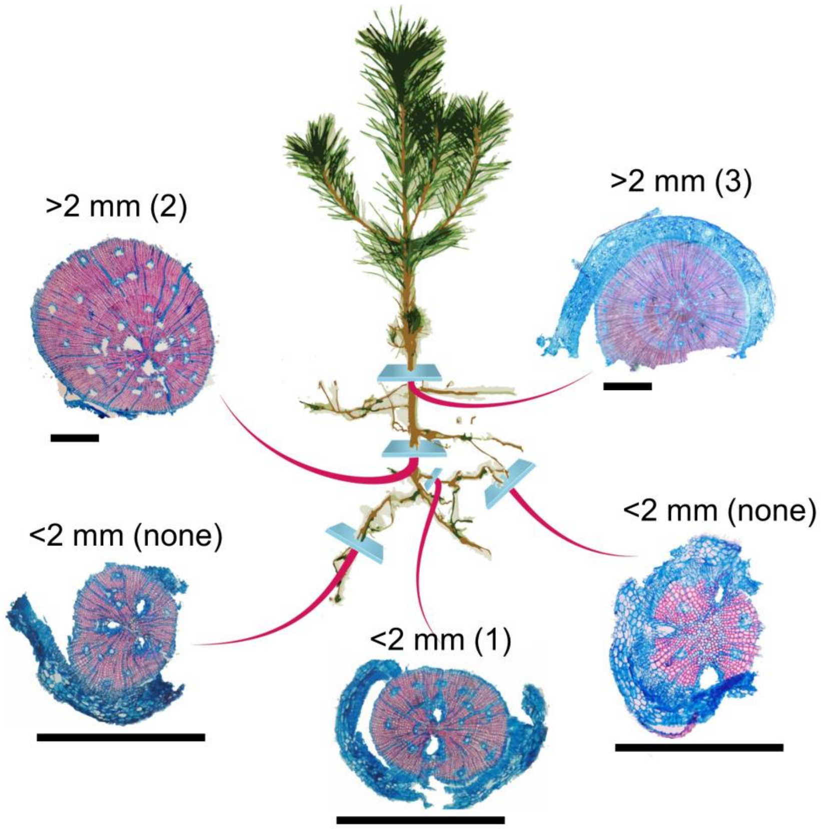

Supplementary Figure 1.

Example of the number of growth rings identified in a three-year old Scots pine (*Pinus sylvestris* L.) seedling to test the robustness of the method. The number of growth rings is given in brackets. The seedling was grown from seeds in the garden of the Swiss Federal Institute for Forest, Snow and Landscape Research WSL. The black line represents the length of 1 mm.

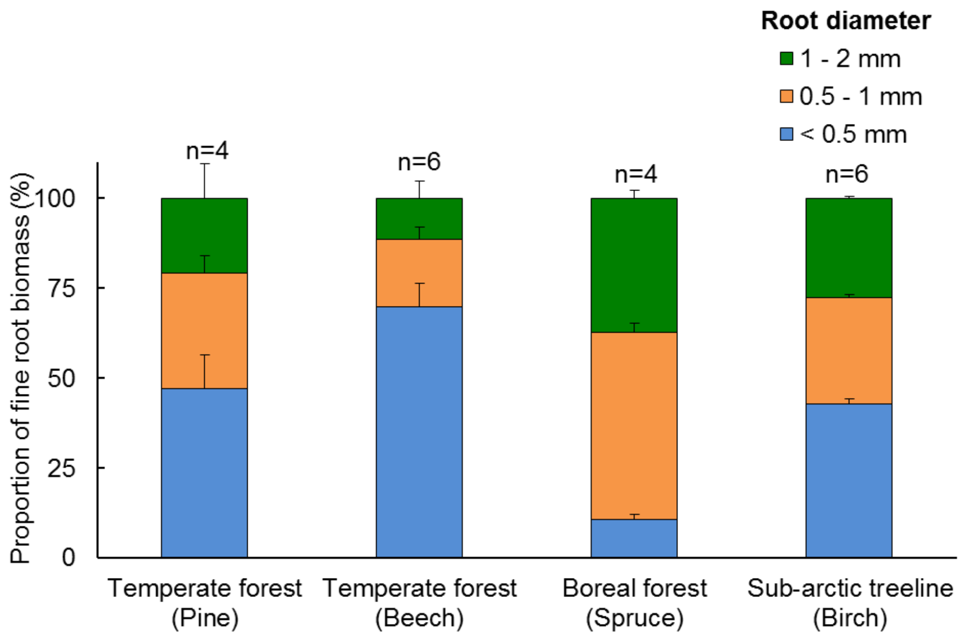

Supplementary Figure 2.

Proportion of fine-root biomass belonging to different diameter classes (mean ± 1SE).

Supplementary Table 1.
Additional environmental characteristics of the four forest sites.

**Forest type**	**Temperate**	**Temperate**		**Boreal**	**Sub-arctic**
Plant species	*Pine*	*Beech*		*Spruce*	*Birch*
	Pfynwald	Schwäbische-Alb	Hainich-Dün	Flakaliden	Tchernaya
Altitude	615 m	460–860 m	285–550 m	310-320 m	200–320 m
Stand basal area	27.3 m^2 ha^{-1}.	18.3-42.5 m^2 ha^{-1}.	29.6 m^2 ha^{-1}.	20 m^2 ha^{-1}	0.8-18.1 m^2 ha^{-1}
Annual mean temperature	9.2 ºC	6.0–7.0 ºC	7.0 ºC	1.2 ºC	-3.9-4.7 ºC
Annual mean precipitation	657 mm	700–1000 mm	500–800 mm	523 mm	450–820 mm
Parent material	Alluvial fan	Jurassic limestone	Loess over Triassic shell limestone	Sandy glacial till	Ultramafic rock
pH *	5.3-6.9	4.5-6.2	7.3	5.0***	4.1-4.5
Soil C:N ratio**	22.3-25.4	12.8-14.3	11.7	31-34***	13.5-21.5

*0-10 cm of the mineral soil. measured in 0.01 mol L^{-1} $CaCl_2$

**0-10 cm of the mineral soil.

***organic layer (pH in H_2O)
