# Supplementary material for: Unravelling the age of fine roots of temperate and boreal forests
Source: Nat Commun. 2018 Aug 1;9:3006. doi: 10.1038/s41467-018-05460-6 (PMC6070616; doi:10.1038/s41467-018-05460-6)
Supplement: Supplementary file 3 — Description of Additional Supplementary Files [file 41467_2018_5460_MOESM3_ESM.pdf]

Description of Additional Supplementary Files

File Name: Supplementary Data 1
Description: Fine root chronological ages, fine root biomasses, fine root radiocarbon values and estimated carbon ages of all samples from different climate zones with different dominant woody species used in this study. This file further contains data on the chronological ages of fine roots grown in in-growth cores and on the chronological ages of roots of tree seedlings. Data on the comparison between chronological ages and estimated carbon ages of the single individual fine roots is also provided in this file.

File Name: Supplementary Software 1
Description: R 3.2.3 code used for the statistical analysis (11 pages).
