# Supplementary material for: Unravelling the age of fine roots of temperate and boreal forests
Source: Nat Commun. 2018 Aug 1;9:3006. doi: 10.1038/s41467-018-05460-6 (PMC6070616; doi:10.1038/s41467-018-05460-6)
Supplement: Supplementary file 4 — Supplementary Software 1 [file 41467_2018_5460_MOESM4_ESM.pdf]

Supplementary Software

Unravelling the age of fine roots of temperate and boreal forests

Solly *et al*.

This file includes:

Supplementary Software 1

Supplementary Software 1

R 3.2.3 code used for the statistical analysis (next 11 pages).

```
setwd("C:/Users/solly/Documents/Manuscript_3RootRings2/For_R")
getwd()

## [1] "C:/Users/solly/Documents/Manuscript_3RootRings2/For_R"

root2<- read.csv("For R2.csv",header=T)
names(root2)

## [1] "Species"          "n"                  "Plot.Tree.number"
## [4] "Diameter..mm."    "Year"               "Age..year."
## [7] "Type"

attach(root2)
str(root2)

## 'data.frame':    40 obs. of  7 variables:
##  $ Species          : Factor w/ 4 levels "Beech","Birch",..: 3 3 3
3 1 1 1 1 1 1 ...
##  $ n                : int  1 2 3 4 6 7 8 9 10 11 ...
##  $ Plot.Tree.number: Factor w/ 20 levels "AEW3","AEW4",..: 15 16
17 18 1 2 3 4 5 6 ...
##  $ Diameter..mm.    : Factor w/ 2 levels "<2","<2 ": 2 2 1 1 1 1 1
1 1 1 ...
##  $ Year             : int  2003 2003 2003 2003 2011 2011 2011 2011
2011 2011 ...
##  $ Age..year.       : num  12 6 14 11.5 19.5 3 7.5 21 14.5 10.5 ..
.
##  $ Type             : Factor w/ 2 levels "Based on 14C",..: 1 1 1
1 1 1 1 1 1 1 ...

#testing the overall difference between root ages based on growth ri
ngs and based on 14C
wilcox.test(Age..year.~Type, paired=TRUE, distribution="exact")

##
##  Wilcoxon signed rank test
##
## data:  Age..year. by Type
## V = 210, p-value = 1.907e-06
## alternative hypothesis: true location shift is not equal to 0

setwd("C:/Users/solly/Documents/Manuscript_3RootRings2/For_R")
getwd()

## [1] "C:/Users/solly/Documents/Manuscript_3RootRings2/For_R"

root<- read.csv("For R.csv",header=T)
names(root)

## [1] "Species"          "n"                  "Plot.Tree.number"
## [4] "Diameter..mm."    "Year"               "F14C"
## [7] "C.age"            "Root.ring.age"      "Difference"

str(root)
```

```
## 'data.frame':    20 obs. of  9 variables:
##  $ Species          : Factor w/ 4 levels "Beech","Birch",..: 3 3 3
3 1 1 1 1 1 1 ...
##  $ n                : int  1 2 3 4 6 7 8 9 10 11 ...
##  $ Plot.Tree.number: Factor w/ 20 levels "AEW3","AEW4",..: 15 16
17 18 1 2 3 4 5 6 ...
##  $ Diameter..mm.    : Factor w/ 2 levels "<2","<2 ": 2 2 1 1 1 1 1
1 1 1 ...
##  $ Year             : int  2003 2003 2003 2003 2011 2011 2011 2011
2011 2011 ...
##  $ F14C             : num  1.15 1.11 1.17 1.14 1.14 ...
##  $ C.age            : num  12 6 14 11.5 19.5 3 7.5 21 14.5 10.5 ..
.
##  $ Root.ring.age    : num  2 1.4 1.8 1 1.8 1.2 1.2 1.7 2.7 2 ...
##  $ Difference       : num  10 4.6 12.2 10.5 17.7 ...
```

```
attach(root)
```

```
## The following objects are masked from root2:
##
##     Diameter..mm., n, Plot.Tree.number, Species, Year
```

```
par(mfrow=c(2,2))
hist(Root.ring.age)
Ring.age<-log10(Root.ring.age)
hist(Ring.age)
hist(C.age)
hist(Difference)
```

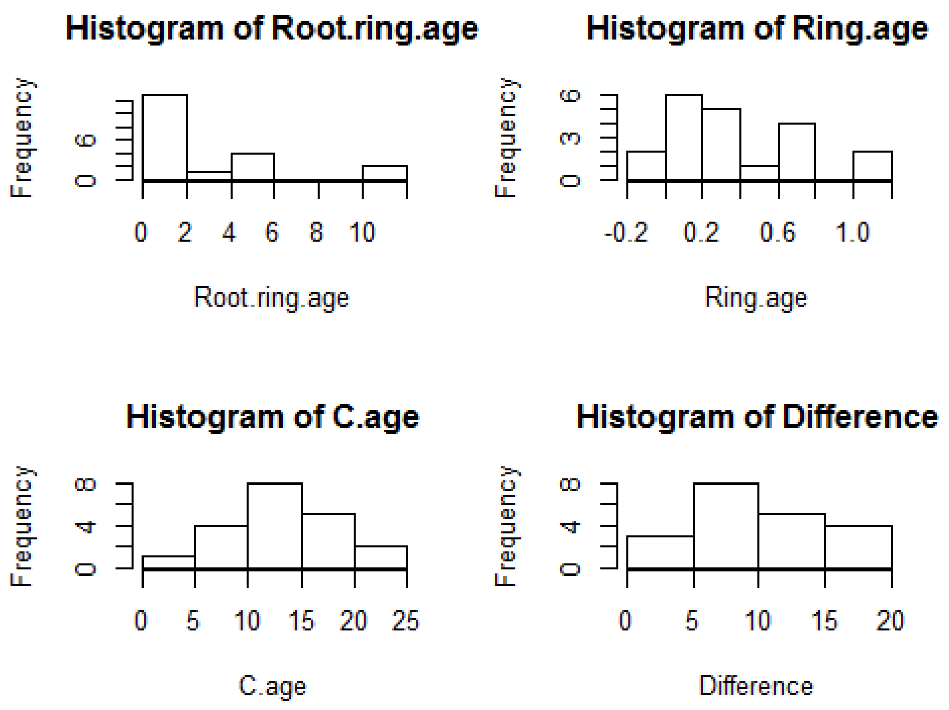


```
#testing the difference between root ages based on growth rings
#accross forests with different dominant woody species
```

```r
anova2<-aov(Ring.age~Species)
summary(aov(anova2))

##             Df Sum Sq Mean Sq F value   Pr(>F)    
## Species      3 1.8520  0.6173   29.52 9.33e-07 ***
## Residuals   16 0.3346  0.0209                     
## ---
## Signif. codes:  0 '***' 0.001 '**' 0.01 '*' 0.05 '.' 0.1 ' ' 1

summary(lm(anova2))

## 
## Call:
## lm(formula = anova2)
## 
## Residuals:
##       Min        1Q    Median        3Q       Max 
## -0.175716 -0.120388  0.007767  0.080110  0.252713 
## 
## Coefficients:
##                Estimate Std. Error t value Pr(>|t|)    
## (Intercept)     0.22941    0.05904   3.886  0.00131 ** 
## SpeciesBirch    0.58976    0.08349   7.064 2.68e-06 ***
## SpeciesPine    -0.05381    0.09335  -0.576  0.57236    
## SpeciesSpruce -0.16473    0.09335  -1.765  0.09669 .  
## ---
## Signif. codes:  0 '***' 0.001 '**' 0.01 '*' 0.05 '.' 0.1 ' ' 1
## 
## Residual standard error: 0.1446 on 16 degrees of freedom
## Multiple R-squared:  0.847,  Adjusted R-squared:  0.8183 
## F-statistic: 29.52 on 3 and 16 DF,  p-value: 9.327e-07

par(mfrow=c(2,2))
plot(anova2)
```

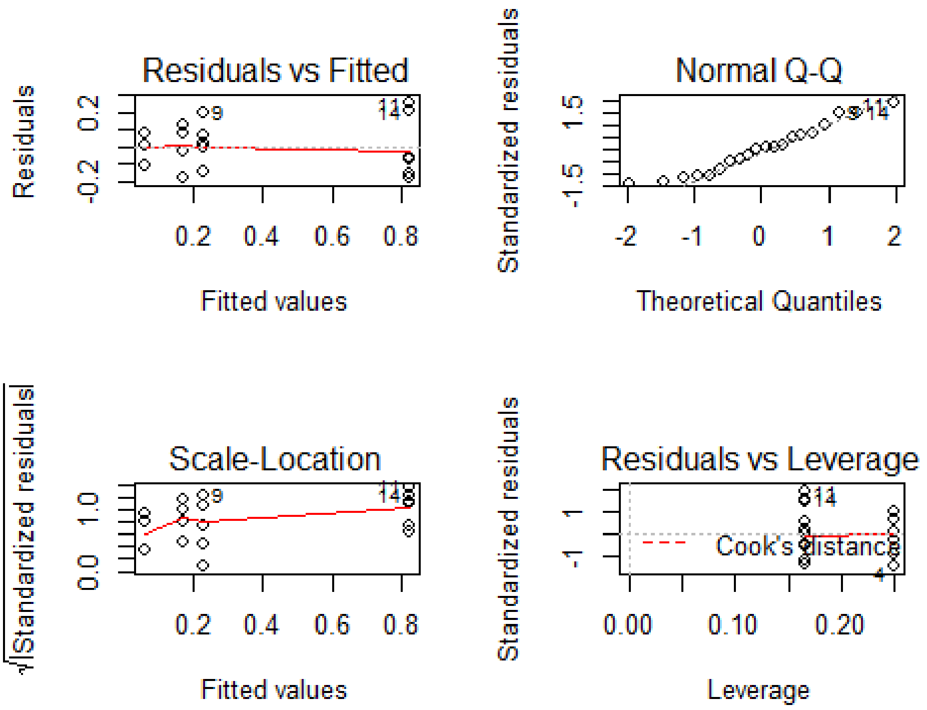


```r
posthoc2 <-TukeyHSD(anova2, which = "Species", conf.level = 0.95)
posthoc2
```

```
##   Tukey multiple comparisons of means
##     95% family-wise confidence level
##
## Fit: aov(formula = Ring.age ~ Species)
##
## $Species
##                      diff        lwr        upr     p adj
## Birch-Beech   0.58975612  0.3508854  0.8286268 0.0000146
## Pine-Beech   -0.05380531 -0.3208709  0.2132603 0.9377166
## Spruce-Beech -0.16472969 -0.4317953  0.1023359 0.3250852
## Pine-Birch   -0.64356143 -0.9106270 -0.3764959 0.0000196
## Spruce-Birch -0.75448581 -1.0215514 -0.4874202 0.0000027
## Spruce-Pine  -0.11092437 -0.4034800  0.1816313 0.7034409
```

```r
#testing the difference between root ages based on 14C
#accross forests with different dominant woody species
anova3<-aov(C.age~Species)
summary(aov(anova3))
```

```
##             Df Sum Sq Mean Sq F value Pr(>F)
## Species      3  263.2   87.73   4.099 0.0246 *
## Residuals   16  342.4   21.40
## ---
## Signif. codes:  0 '***' 0.001 '**' 0.01 '*' 0.05 '.' 0.1 ' ' 1
```

```r
summary(lm(anova3))
```

```
##
## Call:
```

```
## lm(formula = anova3)
##
## Residuals:
##     Min      1Q  Median      3Q     Max
## -9.6667 -2.6750  0.6667  2.1563  8.3333
##
## Coefficients:
##               Estimate Std. Error t value Pr(>|t|)
## (Intercept)     12.667      1.889   6.707 5.04e-06 ***
## SpeciesBirch     6.167      2.671   2.309   0.0346 *
## SpeciesPine     -1.792      2.986  -0.600   0.5569
## SpeciesSpruce   -3.067      2.986  -1.027   0.3197
## ---
## Signif. codes:  0 '***' 0.001 '**' 0.01 '*' 0.05 '.' 0.1 ' ' 1
##
## Residual standard error: 4.626 on 16 degrees of freedom
## Multiple R-squared:  0.4346, Adjusted R-squared:  0.3286
## F-statistic: 4.099 on 3 and 16 DF,  p-value: 0.02456
```

```
par(mfrow=c(2,2))
plot(anova3)
```

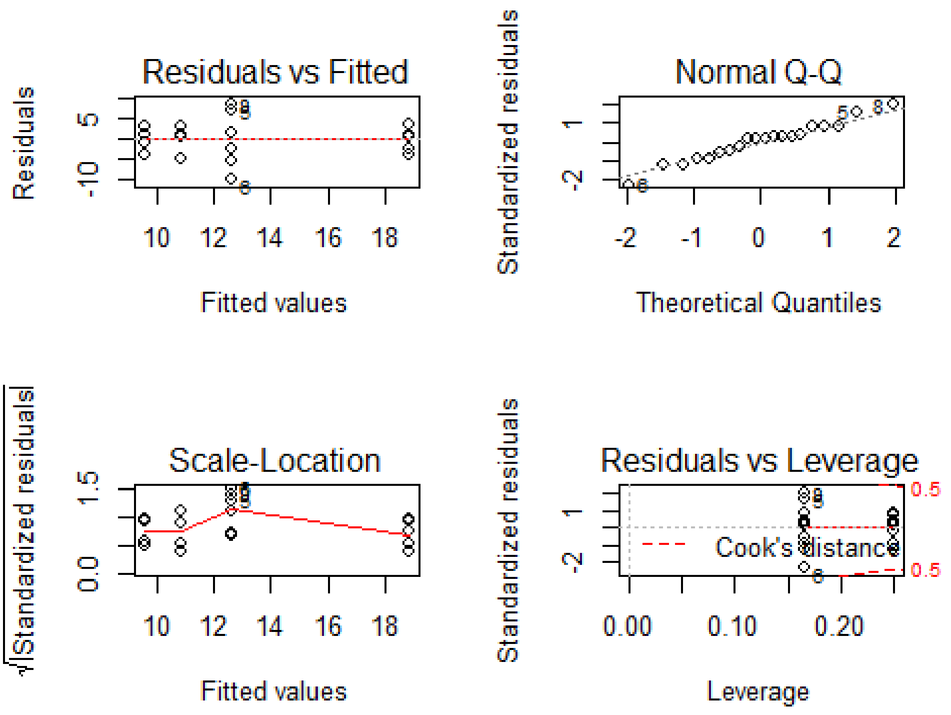


```
posthoc3 <-TukeyHSD(anova3, which = "Species", conf.level = 0.95)
posthoc3
```

```
##   Tukey multiple comparisons of means
##     95% family-wise confidence level
##
## Fit: aov(formula = C.age ~ Species)
##
## $Species
```

```
##                      diff        lwr        upr     p adj
## Birch-Beech   6.166667  -1.475012 13.8083457 0.1374810
## Pine-Beech   -1.791667 -10.335324  6.7519902 0.9305774
## Spruce-Beech -3.066667 -11.610324  5.4769902 0.7365089
## Pine-Birch   -7.958333 -16.501990  0.5853236 0.0724319
## Spruce-Birch -9.233333 -17.776990 -0.6896764 0.0319228
## Spruce-Pine  -1.275000 -10.634107  8.0841072 0.9791935
```

```
#testing the difference between ages yealded from the two methods
#accross forests with different dominant woody species
anova4<-aov(Difference~Species)
summary(aov(anova4))
```

```
##             Df Sum Sq Mean Sq F value Pr(>F)
## Species      3   31.8   10.62   0.478  0.702
## Residuals   16  355.3   22.20
```

```
summary(lm(anova4))
```

```
##
## Call:
## lm(formula = anova4)
##
## Residuals:
##     Min      1Q  Median      3Q     Max
## -9.0936 -2.9562  0.5375  2.9750  8.3912
##
## Coefficients:
##               Estimate Std. Error t value Pr(>|t|)
## (Intercept)     10.912      1.924   5.672 3.47e-05 ***
## SpeciesBirch     0.788      2.721   0.290    0.776
## SpeciesPine     -1.587      3.042  -0.522    0.609
## SpeciesSpruce   -2.487      3.042  -0.818    0.426
## ---
## Signif. codes:  0 '***' 0.001 '**' 0.01 '*' 0.05 '.' 0.1 ' ' 1
##
## Residual standard error: 4.712 on 16 degrees of freedom
## Multiple R-squared:  0.08227,    Adjusted R-squared:  -0.0898
## F-statistic: 0.4781 on 3 and 16 DF,  p-value: 0.702
```

```
par(mfrow=c(2,2))
plot(anova4)
```

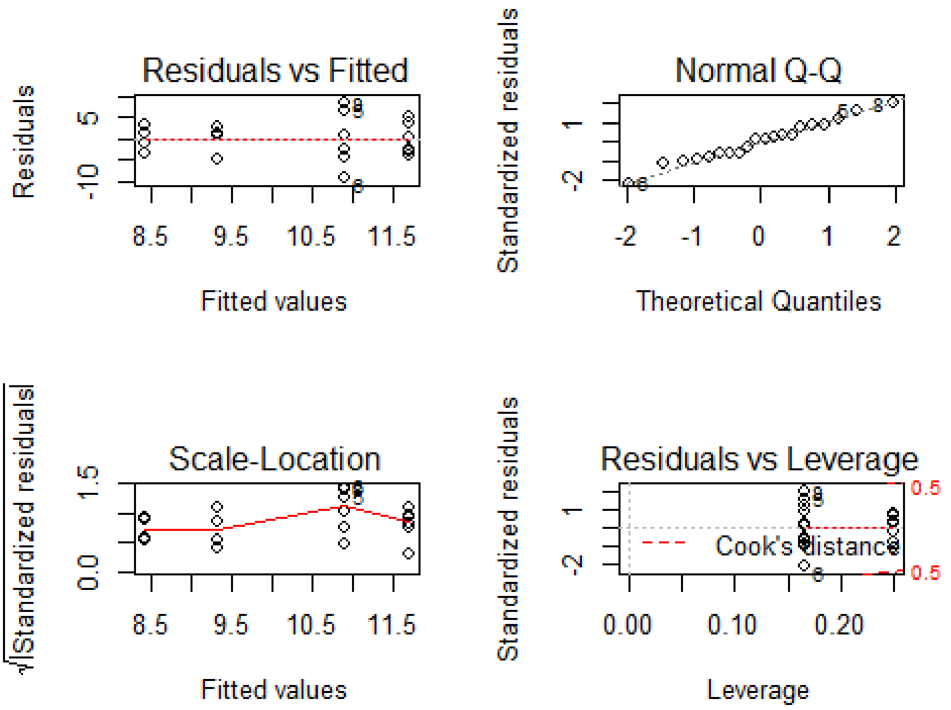


```
posthoc4 <-TukeyHSD(anova4, which = "Species", conf.level = 0.95)
posthoc4

##   Tukey multiple comparisons of means
##     95% family-wise confidence level
##
## Fit: aov(formula = Difference ~ Species)
##
## $Species
##                   diff        lwr      upr     p adj
## Birch-Beech   0.7880481  -6.995692 8.571789 0.9912020
## Pine-Beech   -1.5869519 -10.289438 7.115535 0.9526188
## Spruce-Beech -2.4869519 -11.189438 6.215535 0.8452994
## Pine-Birch   -2.3750000 -11.077487 6.327487 0.8620182
## Spruce-Birch -3.2750000 -11.977487 5.427487 0.7081180
## Spruce-Pine  -0.9000000 -10.433096 8.633096 0.9928318

setwd("C:/Users/solly/Documents/Manuscript_3RootRings2/For_R")
getwd()

## [1] "C:/Users/solly/Documents/Manuscript_3RootRings2/For_R"

root1<- read.csv("For Rdiameters.csv",header=T)
names(root1)

## [1] "Species"          "Plot.Tree.number" "Year"
## [4] "Root.ring.age"    "Diameter.size"

str(root1)

## 'data.frame':    60 obs. of  5 variables:
##  $ Species         : Factor w/ 4 levels "Beech","Birch",..: 3 3 3
3 1 1 1 1 1 1 ...
```

```
##  $ Plot.Tree.number: Factor w/ 20 levels "AEW03","AEW04",..: 15 1
6 17 18 1 2 3 4 5 6 ...
##  $ Year            : int  2003 2003 2003 2003 2011 2011 2011 2011
2011 2011 ...
##  $ Root.ring.age   : num  0.833 1 1 1 1.625 ...
##  $ Diameter.size   : Factor w/ 3 levels "a","b","c": 1 1 1 1 1 1
1 1 1 1 ...
```

```
attach(root1)
```

```
## The following objects are masked from root:
##
##     Plot.Tree.number, Root.ring.age, Species, Year
```

```
## The following objects are masked from root2:
##
##     Plot.Tree.number, Species, Year
```

```
hist(Root.ring.age)
Ring.age<-log10(Root.ring.age)
hist(Ring.age)

#testing the difference between root ages based on growth rings
#between roots with different diameter sizes and forests with differ
ent dominant woody species
anova5<-aov(Ring.age~Diameter.size)
summary(aov(anova5))
```

```
##               Df Sum Sq Mean Sq F value  Pr(>F)
## Diameter.size  2  1.938  0.9689   7.134 0.00176 **
## Residuals     55  7.470  0.1358
## ---
## Signif. codes:  0 '***' 0.001 '**' 0.01 '*' 0.05 '.' 0.1 ' ' 1
## 2 observations deleted due to missingness
```

```
summary(lm(anova5))
```

```
##
## Call:
## lm(formula = anova5)
##
## Residuals:
##      Min       1Q   Median       3Q      Max
## -0.52711 -0.29711 -0.07288  0.26586  0.91982
##
## Coefficients:
##                Estimate Std. Error t value Pr(>|t|)
## (Intercept)      0.1277     0.0824   1.549 0.127109
## Diameter.sizeb   0.2463     0.1165   2.113 0.039141 *
## Diameter.sizec   0.4506     0.1197   3.764 0.000409 ***
## ---
## Signif. codes:  0 '***' 0.001 '**' 0.01 '*' 0.05 '.' 0.1 ' ' 1
##
## Residual standard error: 0.3685 on 55 degrees of freedom
##   (2 observations deleted due to missingness)
```

```
## Multiple R-squared:  0.206,  Adjusted R-squared:  0.1771
## F-statistic: 7.134 on 2 and 55 DF,  p-value: 0.001759

par(mfrow=c(2,2))
```

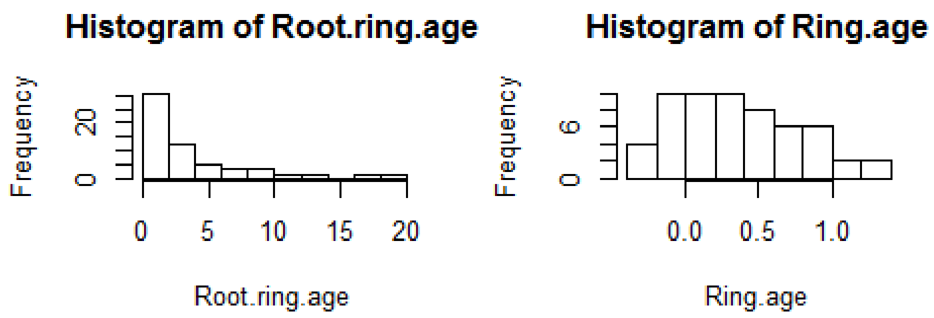


```
plot(anova5)
```

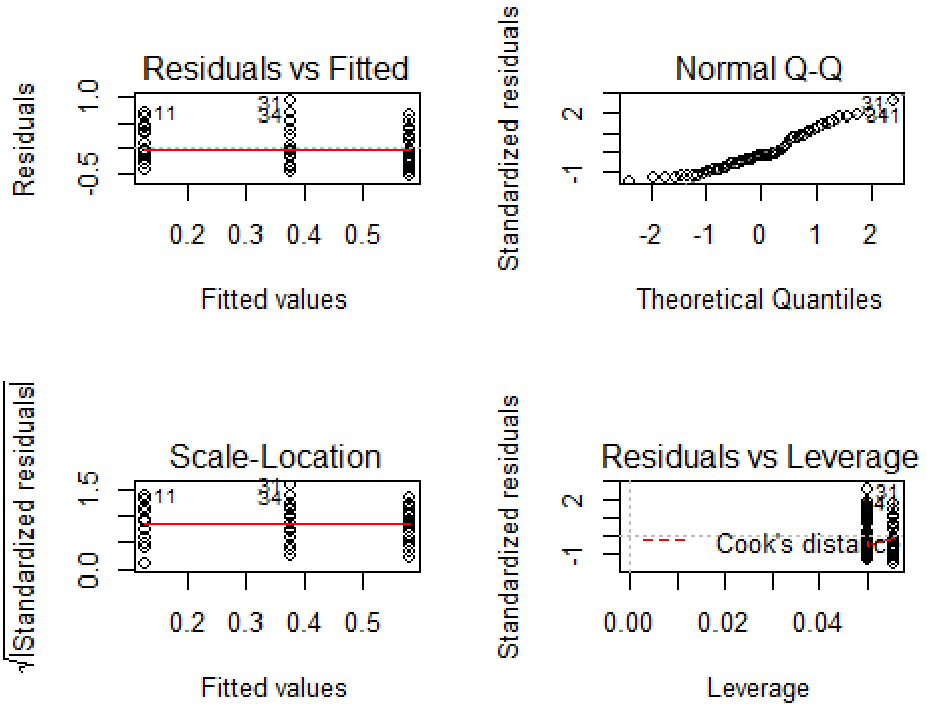


```
posthoc5 <-TukeyHSD(anova5, which = "Diameter.size", conf.level = 0.
95)
posthoc5

##   Tukey multiple comparisons of means
##     95% family-wise confidence level
##
## Fit: aov(formula = Ring.age ~ Diameter.size)
##
## $Diameter.size
##             diff         lwr       upr     p adj
## b-a 0.2462603 -0.03444953 0.5269701 0.0964545
```

```
## c-a 0.4506134  0.16221150 0.7390153 0.0011729
## c-b 0.2043531 -0.08404879 0.4927551 0.2118033
```

```
anova6<-aov(Ring.age~Diameter.size*Species)
summary(aov(anova6))
```

```
##                        Df Sum Sq Mean Sq F value   Pr(>F)
## Diameter.size           2  1.938  0.9689  26.497 2.21e-08 ***
## Species                 3  5.553  1.8511  50.624 1.29e-14 ***
## Diameter.size:Species   6  0.234  0.0390   1.068    0.396
## Residuals              46  1.682  0.0366
## ---
## Signif. codes:  0 '***' 0.001 '**' 0.01 '*' 0.05 '.' 0.1 ' ' 1
## 2 observations deleted due to missingness
```

```
summary(lm(anova6))
```

```
##
## Call:
## lm(formula = anova6)
##
## Residuals:
##      Min       1Q   Median       3Q      Max
## -0.78350 -0.06753 -0.01541  0.09073  0.41108
##
## Coefficients:
##                              Estimate Std. Error t value Pr(>|t|)
## (Intercept)                   0.13608    0.07807   1.743  0.08799
.
## Diameter.sizeb                0.18463    0.11040   1.672  0.10124
## Diameter.sizec                0.32460    0.11579   2.803  0.00738
**
## SpeciesBirch                  0.34639    0.11040   3.138  0.00297
**
## SpeciesPine                  -0.15587    0.12343  -1.263  0.21302
## SpeciesSpruce                -0.40587    0.12343  -3.288  0.00194
**
## Diameter.sizeb:SpeciesBirch   0.21554    0.15613   1.381  0.17410
## Diameter.sizec:SpeciesBirch   0.18177    0.15999   1.136  0.26179
## Diameter.sizeb:SpeciesPine   -0.10344    0.17456  -0.593  0.55639
## Diameter.sizec:SpeciesPine    0.20988    0.18638   1.126  0.26596
## Diameter.sizeb:SpeciesSpruce  0.08826    0.17456   0.506  0.61556
## Diameter.sizec:SpeciesSpruce  0.10224    0.17802   0.574  0.56854
## ---
## Signif. codes:  0 '***' 0.001 '**' 0.01 '*' 0.05 '.' 0.1 ' ' 1
##
## Residual standard error: 0.1912 on 46 degrees of freedom
##   (2 observations deleted due to missingness)
## Multiple R-squared:  0.8212, Adjusted R-squared:  0.7784
## F-statistic: 19.21 on 11 and 46 DF,  p-value: 1.16e-13
```

```
par(mfrow=c(2,2))
plot(anova6)
```

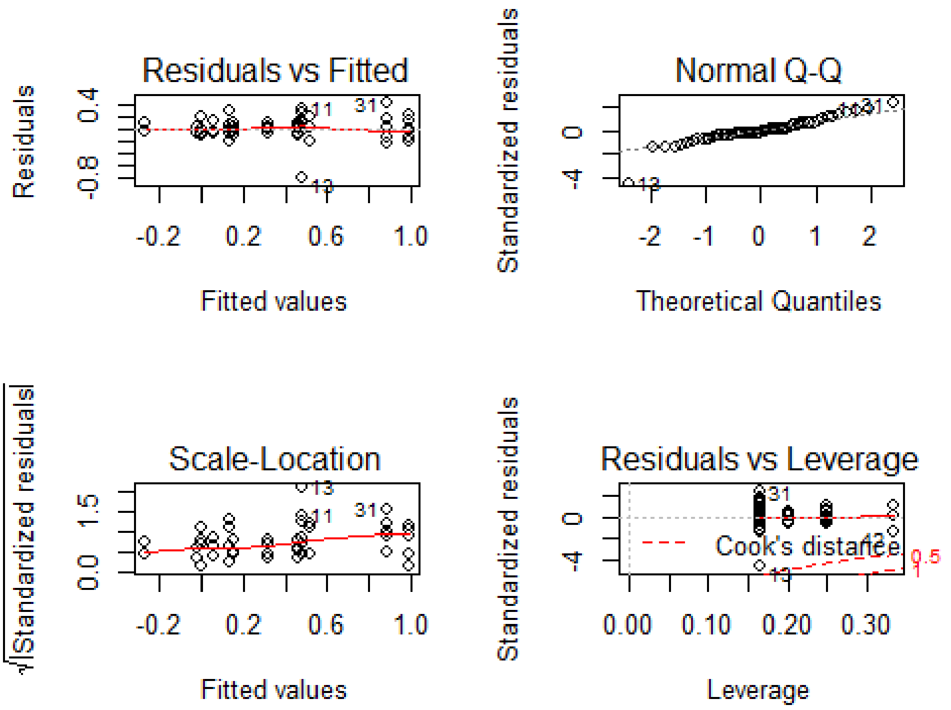


```
posthoc6 <-TukeyHSD(anova6, which = "Species", conf.level = 0.95)
posthoc6

##   Tukey multiple comparisons of means
##     95% family-wise confidence level
##
## Fit: aov(formula = Ring.age ~ Diameter.size * Species)
##
## $Species
##                    diff        lwr           upr     p adj
## Birch-Beech   0.4750956  0.3027154  0.6474758007 0.0000000
## Pine-Beech   -0.1342096 -0.3314392  0.0630200910 0.2800678
## Spruce-Beech -0.3461084 -0.5382839 -0.1539329448 0.0000983
## Pine-Birch   -0.6093052 -0.8043707 -0.4142397132 0.0000000
## Spruce-Birch -0.8212040 -1.0111577 -0.6312503238 0.0000000
## Spruce-Pine  -0.2118988 -0.4246593  0.0008616479 0.0512913
```
